# Early Use of Dinutuximab Beta in Patients with High-Risk Neuroblastoma

**DOI:** 10.1155/2021/6610955

**Published:** 2021-06-19

**Authors:** Neofit Spasov, Mariya Spasova

**Affiliations:** Medical University Plovdiv, Department of Pediatrics and Medical Genetics, Oncohematology Unit, University Hospital “Sveti Georgi”, Plovdiv, Bulgaria

## Abstract

Neuroblastoma is the most common extracranial solid tumor in children, accounting for 15% of all pediatric cancer deaths. High-risk neuroblastoma (HRNB) is a particularly difficult-to-treat form of the disease that requires aggressive multimodality therapy, including induction chemotherapy, consolidation therapy with high-dose chemotherapy and autologous stem cell transplant, and maintenance therapy with dinutuximab beta. Despite treatment advances, the prognosis of these patients remains poor. As a better response to induction therapy has been associated with prolonged survival in patients with HRNB, we hypothesized that early use of dinutuximab beta—post-induction chemotherapy—may improve patient outcomes. We describe here our experience of administering at least one cycle of dinutuximab beta post-induction and prior to surgery in three children with HRNB who did not demonstrate a complete response to induction chemotherapy. All three patients achieved complete remission. Early use of dinutuximab beta may therefore have the potential to improve outcomes in patients with HRNB.

## 1. Introduction

Although neuroblastoma is a rare malignant disease, it is the most common extracranial solid tumor in childhood, accounting for 15% of all pediatric cancer deaths [1]. It is a tumor of the peripheral sympathetic nervous system, which generally affects the abdomen (65%), with most tumors originating from the adrenal gland [1]. Due to its clinical heterogeneity, the treatment of neuroblastoma is based on the risk of the individual patient [2]. Any patient with *MYCN* amplification is classified as high risk, as are patients with metastatic disease who are ≥18 months of age (International Neuroblastoma Staging System (INSS) Stage 4) and those <18 months of age with localized primary tumor and metastases limited to skin, liver, and bone marrow, without bone involvement and an 11q aberration (INSS Stage 4S) [2].

Prior to the introduction of intense multimodal therapy for patients with HRNB, overall survival (OS) was <15% [3]. The International Society of Paediatric Oncology European Neuroblastoma (SIOPEN) group undertook a long-term study to optimize treatment for patients with HRNB (HR-NBL1/SIOPEN) [4]. Intensive induction therapy with rapid COJEC (time-intensive cisplatin, carboplatin, cyclophosphamide, vincristine, and etoposide) followed by high-dose chemotherapy with busulfan/melphalan (BuMel) and autologous stem cell transplant (ASCT) improved the 5-year OS to 54% [4]. According to the HR-NBL1/SIOPEN protocol, only patients with a metastatic complete response (CR) or partial response (PR) following induction therapy, with ≤3 abnormal skeletal areas and no bone marrow disease, should progress to consolidation therapy [4]. SIOPEN demonstrated that two cycles of topotecan, vincristine, and doxorubicin (TVD) improved the response rate in patients not meeting these criteria following COJEC induction [5]. At least 50% of patients with HRNB relapse following the completion of induction and consolidation therapy [6, 7]. The addition of maintenance therapy with dinutuximab beta to this approach increased the 5-year OS in patients with HRNB to over 60% for the first time [6].

Dinutuximab beta is a monoclonal antibody that targets disialoganglioside 2 (GD2), which is ubiquitously overexpressed on neuroblastoma cells [8]. It was approved by the European Medicines Agency in 2017 for the treatment of HRNB in patients aged ≥12 months who have achieved at least a PR to induction chemotherapy and received myeloablative therapy and stem cell transplant and patients with a history of relapsed or refractory neuroblastoma, with or without residual disease [9]. SIOPEN currently recommends dinutuximab beta, administered at 10 mg/m^2^ per day for ten days as a continuous intravenous infusion, as the standard of care for patients with HRNB [10]. In the USA, a similar anti-GD2 antibody, known as dinutuximab, has been approved for the treatment of HRNB in the maintenance setting, based on the results of the Children's Oncology Group (COG) trial ANBL0032 [7, 11]. While recent follow-up data confirmed the long-term survival benefit of dinutuximab in these patients, the magnitude of the benefit had decreased over time due to late relapses [12], indicating the need to identify new treatment approaches to further improve outcomes.

As an improved response to induction therapy has been associated with longer survival in patients with HRNB [5, 13], one way to optimize outcomes might be to improve treatment strategies early in the treatment pathway. We postulated that early use of dinutuximab beta—immediately after induction chemotherapy—may increase the patients' chance of remission and event-free survival (EFS). Here, we report the outcomes of three patients with HRNB treated with dinutuximab beta after induction therapy and before surgery.

## 2. Case Presentations

### 2.1. Patient 1

**Figure 1:**
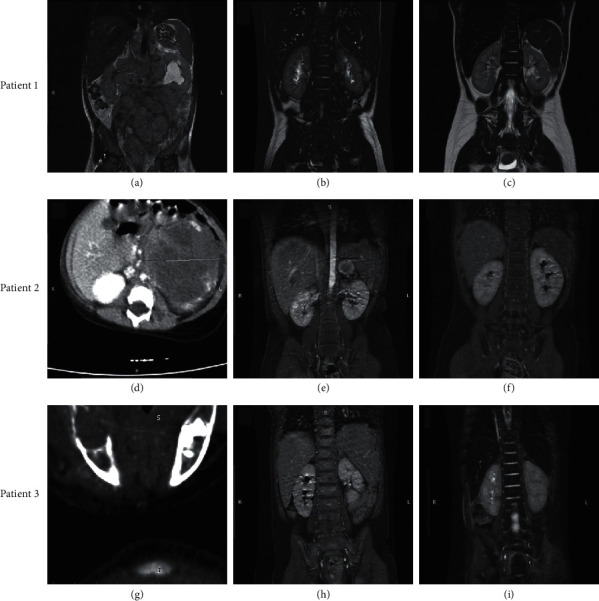
Abdominal MRI scans for Patient 1 (a–c), Patient 2 (d–f), and Patient 3 (g–i). MRI scans for Patient 1: (a) at diagnosis, (b) following induction therapy with eight cycles of COJEC, a cycle of adjuvant dinutuximab beta, and two cycles of adjuvant TVD, and (c) at the end of maintenance therapy with dinutuximab beta. MRI scans for Patient 2: (d) at diagnosis, (e) following induction therapy with eight cycles of COJEC, and (f) following three cycles of dinutuximab beta maintenance therapy. MRI scans for Patient 3: (g) at diagnosis, (h) following induction therapy with seven cycles of COJEC, and (i) following three cycles of second-line immunochemotherapy with irinotecan/temozolomide and dinutuximab beta and surgery of the primary abdominal tumor.

In June 2018, a 2-year and 10-month-old girl was diagnosed with INSS Stage 4 neuroblastoma (Tables 1 and 2). At diagnosis, the primary tumor was located in the left adrenal gland, with metastases in multiple abdominal and pelvic lymph nodes, and central nervous system and bone marrow involvement. Infiltration of the bone marrow was evaluated using trephine biopsy followed by flow cytometry for CD45^−^/CD56^+^ cells in the bone marrow aspirate, a technique commonly used to confirm bone marrow involvement in neuroblastoma [14–16]. A Multiplex Ligation-dependent Probe Amplification (MLPA) analysis of the tumor tissue, carried out in a certified genetic laboratory in Bulgaria (Genica), revealed *MYCN* gene amplification, a hallmark of aggressive neuroblastoma. This analysis is frequently used to detect *MYCN* gene amplification in neuroblastoma [17–19]. Abdominal magnetic resonance imaging (MRI) showed multiple intraperitoneal and retroperitoneal masses of up to 5 cm in size (Figure 1A). The patient had very high tumor marker levels, with serum neuron-specific enolase (NSE) > 370 ng/mL (detection maximum) and urinary homovanillic acid (HVA) and vanillylmandelic acid (VMA) levels 10 times the upper limit of normal (ULN).

The patient received eight cycles of rapid COJEC induction therapy. Abdominal MRI showed shrinkage of the tumors to ≤2 cm, with some showing central necrosis after cycle 4 of COJEC and further tumor shrinkage following cycle 8.

The patient then received neoadjuvant dinutuximab beta 10 mg/m^2^ daily for 10 days, after which NSE, HVA, and VMA levels normalized. During the first course of dinutuximab beta, the patient experienced mild-to-moderate pain, in line with previous reports [9], with no other side effects. Therapy continued with two cycles of TVD. Abdominal MRI undertaken to plan the surgery showed further shrinkage of the tumors (Figure 1B). The primary tumor was totally resected, ten infiltrated lymph nodes were removed, and a left-sided oophorectomy was performed. Tumor necrosis was 100%. Following another cycle of adjuvant TVD, the patient received a second cycle of adjuvant dinutuximab beta 10 mg/m^2^ over 10 days, followed by consolidation therapy with BuMel and ASCT.

After the ASCT, the patient received four cycles of dinutuximab beta 10 mg/m^2^ daily for 10 days as maintenance therapy. Follow-up abdominal MRI showed complete resolution of the disease with no detectable tumors immediately following completion of maintenance therapy (Figure 1C). As of April 2021, 19 months after completing maintenance therapy, the patient is still in full remission according to imaging studies, tumor markers, and clinical status.

### 2.2. Patient 2

Patient 2 is an 8-month-old boy diagnosed with INSS Stage 4, *MYCN* amplified neuroblastoma in August 2019 (Tables 1 and 2). At diagnosis, a CT scan showed the primary tumor in the left adrenal gland, with enlarged para-aortic and paracaval lymph nodes (Figure 1D). Bone marrow trephine biopsy confirmed total bone marrow involvement. Serum NSE level was >370 ng/mL and urinary HVA and VMA levels were five times the ULN.

The patient received eight cycles of rapid COJEC induction therapy. A PET-CT scan after cycle 3 of COJEC showed that the primary tumor was metabolically active and that bone marrow metabolism was increased throughout the body. An abdominal MRI after the completion of induction therapy showed a PR in the primary tumor and local lymph nodes (Figure 1E), and bone marrow trephine biopsy indicated no bone marrow involvement. The patient subsequently received neoadjuvant dinutuximab beta 10 mg/m^2^ daily for 10 days, followed by total resection of the primary tumor and two infiltrated lymph nodes. Tumor necrosis was 98%. A second cycle of immunotherapy with dinutuximab beta 10 mg/m^2^ for 10 days was administered following surgery, after which the patient underwent consolidation therapy with BuMel and ASCT. After the second dinutuximab beta cycle, we evaluated the tumor markers, which were normal. Three cycles of maintenance therapy with dinutuximab beta (10 mg/m^2^ for 10 days) were administered following ASCT. An MRI after the completion of dinutuximab beta maintenance therapy showed no residual tumor in the abdomen (Figure 1F), and bone marrow trephine biopsy and flow cytometry demonstrated no evidence of bone marrow involvement. Similar to Patient 1, this patient also reported no side effects associated with dinutuximab beta, except for mild-to-moderate pain during the first cycle of therapy. As of April 2021, almost 11 months after completing dinutuximab beta maintenance therapy, the patient was still in full remission.

### 2.3. Patient 3

Patient 3 is a 2-year-old girl diagnosed with *MYCN* and *DDX1* amplified, INSS Stage 4, neuroblastoma in December 2019 (Tables 1 and 2). The primary tumor was located in the left adrenal gland, with bone metastasis in the right side of the mandibula and local cervical lymphadenitis (Figure 1G). In addition, trephine biopsy demonstrated total bone marrow involvement.

Following diagnosis, the patient received seven cycles of rapid COJEC induction therapy. A control bone marrow biopsy performed after cycle 4 revealed total regression of the bone marrow infiltration. After cycle 7, the patient developed severe pancytopenia that persisted for more than 20 days, severe peripheral edema, arterial hypertension (220/160 mmHg), severe proteinuria with erythrocytes and leucocytes in the sediment, elevated serum NSE and urine catecholamines, with no bone marrow hemophagocytosis. MRI scans following COJEC showed a reduction in the size of the primary tumor on the left adrenal gland and the metastasis on the mandibula, with full regression in the cervical lymph nodes (Figure 1H). However, both kidneys were 2 cm larger than the size on the initial MRI, without focal lesions and with a normal ratio. Trephine biopsy demonstrated nearly total bone marrow involvement. Thus, the patient had stable disease according to the solid tumor formations in the left adrenal gland and in the mandibula, but with isolated bone marrow progression and secondary paraneoplastic membraneous glomerulonephritis, which was confirmed by biopsy.

The patient was treated with three cycles of second-line immunochemotherapy comprising irinotecan/temozolomide on days 1–5 and dinutuximab beta 10 mg/m^2^ per day for 10 days on days 6–16 of each 21-day cycle. As expected, the patient experienced mild-to-moderate pain during the first cycle of dinutuximab beta treatment but did not report any other side effects during the subsequent two cycles despite the more frequent administration. Cycle 1 and partly cycle 2 were administered with concomitant supportive therapy, after which renal function and blood pressure returned to normal. Following cycle 3 of second-line immunochemotherapy, renal function was normal, as were bone marrow and MRI results (Figure 1I). Serum NSE and urine HVA and VMA levels, which were raised during bone marrow progression, normalized following second-line immunotherapy. Of note, when chemotherapy was followed by immunotherapy in this patient, the aplasia associated with chemotherapy was shorter, with faster hematologic recovery than when chemotherapy was given alone, in our experience.

Following total resection of the primary tumor in the left adrenal gland and radiotherapy of the mandibular metastases, full remission was confirmed, with no evidence of residual tumors and normal bone marrow. The patient then underwent consolidation therapy with BuMel and ASCT and 3 cycles of dinutuximab beta maintenance therapy. As of April 2021, 12 months after surgery, the patient remains in full remission.

## 3. Discussion

HRNB is a difficult-to-treat malignancy that requires multimodal therapy [3]. According to SIOPEN, the standard of care for patients with HRNB is intensive induction therapy with rapid COJEC ± TVD, followed by consolidation therapy with high-dose BuMel and ASCT, and maintenance therapy with dinutuximab beta [4, 5, 10]. However, despite treatment advances over the past decade, the prognosis in children with HRNB remains poor [3]. Therefore, there is a need to further improve treatment strategies for these patients. An improved response to induction therapy has previously been associated with longer survival in patients with HRNB [5, 13], suggesting that strategies to optimize treatment early in the treatment pathway may be of particular value. Indeed, encouraging results were found in a retrospective case series of patients with newly diagnosed HRNB treated concomitantly with induction chemotherapy and dinutuximab, an anti-GD2 antibody similar to dinutuximab beta that has been approved for the treatment of HRNB in the USA [20]. All six patients tolerated the treatment without significant toxicity, completed induction therapy without disease progression, and demonstrated clinical benefits [20]. In addition, results of a phase II trial of the humanized anti-GD2 antibody hu14.18K322A demonstrated that adding this anti-GD2 antibody to induction chemotherapy in patients with newly diagnosed HRNB significantly improved early responses, reduced tumor volumes, and achieved an encouraging 2-year EFS [21]. Therefore, early treatment with dinutuximab beta might also be beneficial, not only in patients with relapsed or refractory NB, as demonstrated previously, but also in those with newly diagnosed HRNB [22].

We report here our experience of administering one cycle of dinutuximab beta post-induction and prior to surgery in three patients with HRNB who did not achieve a CR with induction therapy. While the patients were treated with different protocols, all patients received at least one cycle of dinutuximab beta prior to surgery. All three protocols improved responses, resulting in complete remission. The early use of immunotherapy also led to improved tumor necrosis and normalized tumor markers prior to surgery, both of which would potentially improve OS and EFS in these patients. Furthermore, in Patient 3, early disease progression following induction therapy was successfully treated with second-line chemotherapy plus dinutuximab beta, suggesting that dinutuximab beta has the potential to be used to overcome paraneoplastic events.

Despite dinutuximab beta being administered using different regimens, the patients only reported mild-to-moderate pain during the first cycle of therapy, which is in line with its known safety profile [9], with no other side effects reported. Therefore, the frequent administration of dinutuximab beta did not lead to an increase in side effects. As of April 2021, all three children were still in complete remission and being closely followed.

In conclusion, early dinutuximab beta given immediately post-induction chemotherapy or, in case of progressive disease, during induction in combination with second-line chemotherapy may be a promising strategy to improve responses in patients who do not achieve a CR with induction chemotherapy, potentially improving their OS and EFS. Further studies are needed to evaluate the efficacy and tolerability of early dinutuximab beta use in patients with HRNB, including combining induction chemotherapy with immunotherapy not only with irinotecan/temozolomide, which was well tolerated in Patient 3, but also with more myeloablative agents.

## Figures and Tables

**Table 1:** Key details of the patients' diagnosis, treatment, and outcome.

	Patient 1	Patient 2	Patient 3
Age at diagnosis	2 years and 10 months	8 months	2 years

Date of diagnosis	04 June 2018	04 September 2019	18 December 2019

INSS stage	4	4	4

MYCN status	*MYCN* amplified	*MYCN* amplified	*MYCN* and *DDX1* amplified

Primary tumor	Left adrenal gland	Left adrenal gland	Left adrenal gland

Metastases	(i) Multiple enlarged abdominal and pelvic lymph nodes (ii) Solid CNS lesions and leptomeningeal thickening (iii) BM involvement	(i) Enlarged para-aortic and paracaval lymph nodes (ii) BM involvement	(i) Metastasis in mandibula (ii) Enlarged cervical and tonsilar lymph nodes (iii) BM involvement

Treatment	(i) 8 cycles of COJEC (ii) 1 cycle of DB (iii) 2 cycles of TVD (iv) Surgery (v) 1 cycle of TVD (vi) 1 cycle of DB (vii) BuMel + ASCT (viii) 4 cycles of DB	(i) 8 cycles of COJEC (ii) 1 cycle of DB (iii) Surgery (iv) 1 cycle of DB (v) BuMel + ASCT	(i) 7 cycles of COJEC (ii) 3 cycles of I/T and DB^∗^ (iii) Surgery (iv) BuMel + ASCT

Current status	Complete remission	Complete remission	Complete remission

^∗^I/T was administered on days 1−5 and DB on days 6−16 of each 21-day cycle. ASCT: autologous stem cell transplant; BM: bone marrow; BuMel: busulfan and melphalan; CNS: central nervous system; COJEC: cisplatin (C), vincristine (O), carboplatin (J), etoposide (E), and cyclophosphamide (C); DB: dinutuximab beta; D: doxorubicin; INSS: International Neuroblastoma Staging System; I/T: irinotecan/temozolomide; T: topotecan; V: vincristine.

**Table 2:** Key details of the patients' disease at different stages of treatment.

	At diagnosis	After COJEC	After DB
Patient 1	(i) Primary tumor in left adrenal gland with metastases in multiple abdominal and pelvic lymph nodes (ii) Intra- and retroperitoneal masses ≤5 cm on MRI (iii) CNS and BM involvement (iv) Very high tumor marker levels (NSE, HVA, VMA)	(i) Shrinkage of tumors to ≤2 cm on MRI (ii) Total response according to BM involvement (iii) PR according to CNS involvement	After 1 cycle of DB and 2 cycles of TVD: (i) Further shrinkage of tumors on MRI (ii) NSE, HVA, and VMA levels normalized (iii) No evidence of leptomeningeal thickening and solid CNS metastases on CNS MRI (iv) No BM involvement After surgery: (i) 100% tumor necrosis After 1 cycle of TVD, 1 cycle of DB, BuMel + ASCT, and 4 cycles of DB: (i) No residual tumors in the abdomen and no signs of leptomeningeal thickening on MRI (ii) No BM involvement (iii) Patient in full remission

Patient 2	(i) Primary tumor in left adrenal gland with enlarged para-aortic and paracaval lymph nodes (ii) Total BM involvement (iii) High tumor marker levels (NSE, HVA, VMA)	(i) PR in primary tumor and local lymph nodes on MRI (ii) No BM involvement	After 1 cycle of DB and surgery: (i) 98% tumor necrosis After another cycle of DB: (i) NSE, HVA, and VMA levels normalized After BuMel + ASCT and 3 cycles of DB: (i) No residual tumors on MRI (ii) No BM involvement (iii) Patient in full remission

Patient 3	(i) Primary tumor in left adrenal gland with bone metastasis on mandibula and local cervical lymphadenitis (ii) Total BM involvement (iii) High tumor marker levels (NSE, HVA, VMA)	(i) Patient developed severe pancytopenia due to BM progression accompanied by secondary paraneoplastic membraneous glomerulopathy (ii) Higher HVA and VMA than those at diagnosis (iii) Shrinkage of primary tumor and metastasis on mandibula and full regression in cervical lymph nodes on MRI (iv) Both kidneys were 2 cm larger than on the initial MRI, without focal lesions and with a normal ratio (v) Nearly total BM involvement	After 3 cycles of DB and I/T: (i) Size of kidneys and renal function normalized (ii) No BM involvement (iii) Tumor markers normalized After surgery and radiotherapy: (i) No residual tumors on MRI After BuMel + ASCT and another 3 cycles of DB: (i) Patient in full remission according to tumor markers, trephine biopsy, CNS and abdominal MRI, and DOTA

ASCT: autologous stem cell transplant; BM: bone marrow; BuMel: busulfan and melphalan; CNS: central nervous system; COJEC: cisplatin (C), vincristine (O), carboplatin (J), etoposide (E), and cyclophosphamide (C); D: doxorubicin; DB: dinutuximab beta; DOTA: dodecanetetraacetic acid; HVA: homovanillic acid; I/T: irinotecan/temozolomide; MPLA: Multiplex Ligation-dependent Probe Amplification; MRI: magnetic resonance imaging; NSE: serum neuron-specific enolase urinary; PR: partial response; T: topotecan; V: vincristine; VMA: vanillylmandelic acid.

## Data Availability

The data that support the findings of this study are available from the corresponding author upon reasonable request.
